# Removal of Cadmium and Chromium by Mixture of Silver Nanoparticles and Nano-Fibrillated Cellulose Isolated from Waste Peels of Citrus Sinensis

**DOI:** 10.3390/polym13020234

**Published:** 2021-01-12

**Authors:** Neha Tavker, Virendra Kumar Yadav, Krishna Kumar Yadav, Marina MS Cabral-Pinto, Javed Alam, Arun Kumar Shukla, Fekri Abdulraqeb Ahmed Ali, Mansour Alhoshan

**Affiliations:** 1 School of Nano Sciences, Central University of Gujarat, Gandhinagar 382030, India; tavker.gini@gmail.com; 2 School of Lifesciences, Jaipur National University, Jaipur 302017, India; yadava94@gmail.com; 3 Institute of Environment and Development Studies, Bundelkhand University, Kanpur Road, Jhansi 284128, India; envirokrishna@gmail.com; 4 Geobiotec Research Centre, Department of Geosciences, University of Aveiro, 3810-193 Aveiro, Portugal; 5 King Abdullah Institute for Nanotechnology, King Saud University, P.O. Box-2455, Riyadh 11451, Saudi Arabia; ashukla@ksu.edu.sa (A.K.S.); mhoshan@ksu.edu.sa (M.A.); 6 Chemical Engineering Department, College of Engineering, King Saud University, P.O. Box-2455, Riyadh 11451, Saudi Arabia; falhulidy@ksu.edu.sa

**Keywords:** citrus sinensis, nano-fibrillated cellulose, silver nanoparticles, acid hydrolysis, heavy metal sorption

## Abstract

Nano-fibrillated cellulose (NFC) was extracted by a chemical method involving alkali and acid hydrolysis. The characterisation of the citrus sinensis fruit peel bran and nano-fibrillated cellulose was performed by XRD, FTIR, TEM, and FESEM. XRD confirmed the phase of NFC which showed monoclinic crystal with spherical to rod shape morphology with a size of 44–50 nm. The crystallinity index of treated NFC increased from 39% to 75%. FTIR showed the removal of lignin and hemicellulose from waste peels due to the alkaline treatment. Silver nanoparticles were also synthesised by utilizing extract of citrus sinensis skins as a reducing agent. Pharmaceutical effluent samples from an industrial area were tested by Atomic Absorption Spectrometry. Out of the four metals obtained, cadmium and chromium were remediated by silver nanoparticles with nano-fibrillated cellulose via simulated method in 100 mg/L metal-salt concentrations over a time period of 160 min. The highest removal efficiency was found for cadmium, i.e., 83%, by using silver and NFC together as adsorbents. The second highest was for chromium, i.e., 47%, but by using only NFC. The Langmuir and Freundlich isotherms were well fitted for the sorption of Cd (II) and Cr (II) with suitable high R^2^ values during kinetic simulation. Thus, the isolation of NFC and synthesis of silver nanoparticles proved efficient for heavy metal sorption by the reuse of waste skins.

## 1. Introduction

The fruit of the citrus species Citrus × sinensis is sweet orange. Orange peel is a waste by-product from fruit juice factories across the world. All the plant matter constitutes about 30% cellulose; cotton and wood being the highest, i.e., 90% and 50%, respectively [1]. The polysaccharide available in abundance having the repetitive unit (C_6_H_10_O_5_)_n_ and comprising of D-glucose is cellulose. This cellulose, being a fibrillary constituent of a plant cell, can be extracted from numerous natural sources such as wood and a few lignocellulosic fibres. There are chains that exist in cellulose which are stacked in an ordered format to make up a compact microfibril, which can be stabilized by hydrogen bonding whether it is inter-nuclear or intra-nuclear. Materials with cellulosic content have been investigated by researchers for decades due to their easy surface modifications and wide applications [2,3]. The existence of cellulose that is found as a common material in plant cell walls was first acknowledged by Anselm Payen in 1838, and this cellulose occurs in almost its purest form in the fibres of cotton. Yet, in wood, stalks and plant leaves, it is found in combination with lignin and hemicelluloses [4,5]. As a function of plant species along with its growth function, crystalline and amorphous domains are obtained in native cellulose fibres in variable ratios. Thus, it makes the properties of cellulose nanocrystal widely dependent on the sources of cellulose. The extraction, finding a suitable application, and its characterisation have given rise to varied terms, crystallites, whiskers, nanocrystals, nanofibers and nanofibrils, that have generated much activity globally: (i) NCC (NanoCrystalline Cellulose) and (ii) NFC (Nano-fibrillated Cellulose). Some of the novel methods for cellulose production include top-down methods that involve physical/enzymatic/chemical techniques for its isolation from agricultural/forest residue and wood, while bottom-up methods involve glucose bacteria to develop nano-fibrillated cellulose [6,7]. These cellulosic materials with one of their dimensions in the nanometre range are termed generically as nanocelluloses. Nano-fibrillated Cellulose (NFC) pertains to fibres that have been fibrillated to accomplish agglomerates of cellulose microfibril units; they are less than 100 nm in diameter with a length of several micrometres. Several terminologies exist for relating this material, but most often Nano/MicroFibrillated Cellulose (NFC/MFC) is used [8]. Numerous approaches can deliver cellulose nanofiber extraction, leading to diverse kinds of nanofibrillar materials, which depends on the raw material, pre-treatment, and disintegration of cellulose chains [9,10,11,12,13]. Since the citrus sinensis consists of a considerable amount of cellulose (14%), this material is potentially appropriate as a reinforcing component in high-performance composites. NFC can be produced by chemical or mechanical treatments such as acid hydrolysis. During the acid hydrolysis, chemistry for the hydrolytic cleavage of the glycosidic bonds takes place chiefly in the amorphous sections of the cellulose, which releases individual crystallites [14].

Bai and co-workers described a technique for the production of nanocrystalline cellulose that makes use of a narrow size distribution. The isolation and characterisation of cellulose obtained from sugar cane bagasse was reported by Sun et al. [15]. Wood definitely comprises of cellulose, but non-wood sources such as stems and leaves [16], cotton [17], sea animals [18], and sugar beet [19] have been used recently as raw materials to separate cellulose nanofibrils by chemical methods. Coconut husk fibres were used to prepare cellulose nanofibres by Imam et al. Gas-phase surface esterification of cellulose microfibrils and whiskers has been reported by Berlioz et al. Microfibrillated cellulose from the skins of prickly pear fruit was developed by Habibi et al. [20].

Metal nanoparticles constitute an important part of nanotechnology where numerous applications, depending on their tuneable chemical and physical properties, have been studied. Various capping agents have been utilized for synthesizing silver from silver nitrate, including chemical substrates and plant origins via top-down and bottom-up approaches. Researchers have made use of stem extracts, medicinal plants and alcoholic extracts and achieved different morphology and size [21,22].

Pharmaceutical industries generate compounds of waste containing organic and heavy metals which contaminate soil and water, and this is a major problem faced by the world [23]. Nanomaterials have been displaying faster rate kinetics in water treatment and also higher efficiency due to their high specific surface area and huge number of unsaturated atoms on their surfaces [24]. These advantages lead to an enhancement in adsorption capacity for the removal of organic and inorganic pollutants. The cleansing of toxic metals from wastewater by utilizing agricultural waste, based on the sorption phenomenon, has been considered a favourable technology. However, current research indicates that the use of agricultural waste can have certain drawbacks, such as the inclusion of colour, odour, lower sorption capacity, etc., restricting their commercial use. Hence, the search for cost-effective biomaterials with efficient and enhanced sorption along with their stability to use a minimal biomass dose is in great demand. Assorted inorganic nano-structured materials have been explored for remediating metallic ions but were recently associated with toxicity issues. One way to address such issues related to sustainability is to incorporate renewable materials of miniaturized elements of plant origin [25]. The skill to control, design, and manipulate organic materials on the nano scale to minimize contaminants and simultaneously avoid environmental risk is a major challenge for our 21st century.

In the previous literature reports, various types of cellulosic resources were used as precursors for the generation of nanocellulose. There are rarely any reports where metal nanoparticles have been used in combination with isolated polymers from discards and applied in simulated heavy metal removal. The removal of any heavy metals or any pollutant is executed by expensive clays, synthetic polymers and catalysts containing metals, which have costly precursors. Thus, as a novelty, we chose seasonal citrus sinensis peels grown in West India as the starting material for natural polymer extraction and also as an extract for reducing the nitrate of silver to obtain silver nanoparticles. Thus, waste as a discard can treat the effluent waste if applied at a commercial level with biodegradable polymers along with an economic route. The main objective was value addition to this agricultural waste, which is at present thrown away after use in juice shops or factories. This will open new avenues for using such abundant, renewable, and inexpensive agrowaste materials for developing value-added products.

## 2. Experimental Section

### 2.1. Materials

Analytical grades of sodium hydroxide, sodium hypochlorite, glacial acetic acid, hydrochloric acid, sulphuric acid and silver nitrate from Sigma Aldrich, India were used. The orange waste peels were obtained from juice vendors residing and selling by street side at Gandhinagar, Gujarat, India.

### 2.2. Extraction of Nano-Fibrillated Cellulose (NFC)

#### 2.2.1. Bran Preparation

Waste peels of oranges were washed and immediately rinsed in 1% *w*/*v* potassium meta bisulphite solution for 24 h to inhibit oxidation. The rinsed peels were dried in a hot air oven at 60 °C for 24 h. After drying, the peels were crushed in a grinder and the finest particles were taken and sieved through 70 mesh screen. The bran was stored at 4 °C in sealed containers. The total yield of bran obtained was found to be 52.10%.

#### 2.2.2. Chemical Treatment

**Figure 1:**
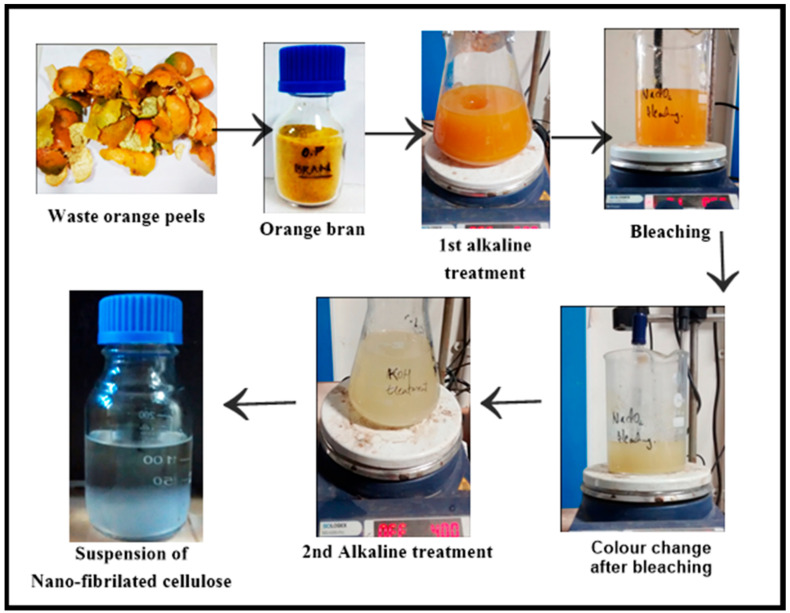
Steps involved in isolation of nano-fibrillated cellulose (NFC).

The method for isolation was similar to the one described by Pelissari M. and co-workers [23]. In 5% KOH solution, the bran (8 gm) was kept under mechanical stirring at room temperature for 16 h. After the alkaline treatment, the insoluble residue was bleached with NaClO_2_ solution for 1 h at 70 °C, pH 5.0 which was adjusted with 10% acetic acid. The residue was neutralised, washed and centrifuged at 6000 rpm for 20 min at 25 °C. Again, the second alkaline treatment was repeated with 5% KOH. The insoluble residue was subjected to acid hydrolysis for an hour with 1% H_2_SO_4_ at 80 °C. The final residue was neutralised, washed and centrifuged as performed in the earlier step and the diluted suspension was kept in a sealed container at 4 °C. The suspension of cellulose was dried by lyophilising and stored at 4 °C and was denoted as NFC. The protocol was similar to that followed by our previous reports [26,27]. The images captured at specific steps are depicted in Figure 1.

### 2.3. Synthesis of Silver Nanoparticles (Ag-NP)

A total of 10 g of waste citrus sinensis peels was washed and boiled in 100 mL distilled water. Further, the peels were crushed and the extract was filtered through Whatman filter paper 1. An aqueous solution (2 mM) of silver nitrate (AgNO_3_) was prepared and used for the synthesis of silver nanoparticles. A total of 6 mL of extract was added to 80 mL of 2 mM AgNO_3_ solution at room temperature at acidic pH 4. The reduction of silver nitrate to silver ions was confirmed by the colour change from colourless to brown when kept in the dark for 12 h. The solution that was reduced underwent centrifugation at 6000 rpm for 20 min. The supernatant liquid was discarded but the pellet obtained was re-dispersed in de-ionized water. This was repeated several times to rinse and remove the absorbed substances on the surface of the silver nanoparticles. The suspension was dried at 60 °C for 3 h to attain black powdered nanoparticles and used further for spectroscopic determination to assure its formation. The sample was noted by AgNPs.

### 2.4. Sorption Studies

Samples of effluent were taken from pharmaceutical industry at Vatva, GIDC phase—IV. The levels of heavy metals present in them were determined by Atomic Absorption Spectroscopy (AAS). Three inlet sites (E1 E2 E3) and 1 outlet (E4) were chosen.

**Figure 2:**
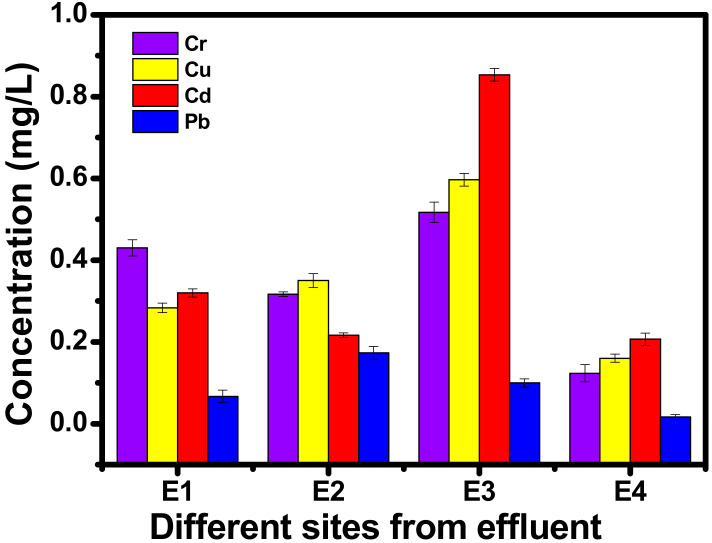
Graphical representation of the estimated heavy metals from pharmaceutical effluent.

Batch experiments were performed by opting for a simulated technique rather than extracting the inorganic metals from the effluents directly. Higher amounts of cadmium (Cd) and chromium (Cr) were detected amongst copper and lead as shown in Figure 2. So, Cd (II) and Cr (II) metal solutions (10 mg/L) were taken separately and adsorption experiments were carried out further. Three sets of each were prepared for biosorption, one having synthesized Ag nanoparticles, the other having NFC, and the third containing both. The pH of the solutions was kept 6.5 using 0.1 M HCl and 0.1 M NaOH, and 0.3 g NFC was taken in each single set up. Metal-loaded biomaterial was transferred to glass bottles with lid and shaken with 100 mL of each desorption reagent as a function of time (10, 20, 40, 80, 160) at room temperature. At each interval, suspensions were stirred and filtered using Whatman 42 filter paper and an estimation of metal ion concentration was carried out by AAS. The metal concentration retained in the solution was computed using Equation (1) and the sorption efficiency for the metal was analysed by Equation (2).

(1) $$Q_{e}\;=\;\left(C_{i}-C_{f}\right)\;\times \;V/W$$

(2) $$\%\;\mathrm{Metal}\;\mathrm{sorption}\;=\;(C_{i}\;-\;C_{f})/C_{i}\;\times \;100$$

where concentration at the beginning and at equilibrium is denoted by *C_i_* and *C_f_*, respectively, and the mass (g) of adsorbent noted by *W* and *V* is the volume (L) contained in the flask. The sorption efficiencies of silver nanoparticles (Ag), cellulose derived from discard (NFC), and the combination of silver nanoparticles with NFC have been compared for the removal of Cd (II) and Cr (II).

#### Adsorption Isotherms

To demonstrate the adsorption by AgNPs, NFC, and Ag + NFC, Freundlich isotherm was utilized for cadmium and chromium heavy metals, for which the equation is given as:

*q* = *K_f_C_e_*^1/*n*^ (3)

The respective data were fitted into the logarithmic equation form:

(4) $$\mathrm{Log}\;q\;=\;\mathrm{Log}\;K_{f}+\frac{1}{n}\;\mathrm{Log}\;C_{e}$$

Here, *q* is the metal sorption taking place per unit mass of the adsorbent, *C_e_* is the residual concentration of the simulated solution of metal ions, and *K_f_* and *n* are constants. *K_f_* is the biosorption capacity while (1/*n*) is the biosorption intensity, and these were calculated from the slope and intercept plotted from Equations (3) and (4).

The general form of Langmuir adsorption equation is given by:

(5) $$\frac{C_{e}}{q_{e}}=\frac{1}{Q_{0}b}+\;\frac{Ce}{Q_{0}}\;$$

Equilibrium concentration is denoted by *C_e_*, while the amount adsorbed by chromium and cadmium is *q_e_*. Two Langmuir constants, *b* and *Q*_0_, are in relation to the energy of adsorption and adsorption capacity, respectively. The linear plot of *C_e_*/*q_e_* vs. *C_e_* infers that the adsorption of heavy metals obeyed the Langmuir model [28,29].

### 2.5. Characterisation of Nano-Fibrillated Cellulose and Silver Nanoparticles

#### 2.5.1. Ultraviolet Spectroscopy (UV)

The silver nanoparticles synthesised were characterised after 24 h with the help of a UV–VIS spectrometer (Dynamica Halo DB-30). The silver nanoparticle solution was diluted and sonicated for 20 min and thus absorbance spectra were obtained. A resolution of 1 nm between 300 and 700 nm possessing a scanning speed of 300 nm/min was used.

#### 2.5.2. Field Emission Scanning Electron Microscopy (FESEM)

The morphology and structure of synthesised NFC can be analysed under a Field emission scanning electron microscope. The Nova Nano FEG-SEM 450 was used throughout the experiment at an accelerating voltage of 15 KV. The lyophilised sample was mounted with carbon tape on the sample holder. Each sample was coated with gold using a sputtering technique. Similarly, the silver nanoparticles were also analysed by FESEM Hitachi SU9000 UHR.

#### 2.5.3. Fourier-Transform Infrared Spectroscopy (FTIR)

FTIR spectra were recorded on a spectrophotometer (IR affinity 1S, Shimadzu). Samples of orange peel bran and cellulose nanoparticles were finely ground and mixed with potassium bromide. The mixture was compressed to pellet form and analysis was performed in the range of 400–4000 cm^−1^.

#### 2.5.4. Transmission Electron Microscopy (TEM)

The size of NFC was analysed using Transmission Electron Microscopy (TEM). Ultra-thin units of isolated nanocellulose were cut by microtone at about −100 °C to allow electrons to pass, and the images were taken by JEM 2010 with an acceleration voltage of 200 kV. A total of 1 mg NFC was suspended into ethanol and ultrasonicated for 5 min.

#### 2.5.5. X-ray Diffraction (XRD)

X-ray diffraction was used to determine the crystallinity of the cellulose based material obtained. Each material was placed on a sample holder and levelled to obtain uniform X-ray exposure. Samples were analysed using an X-ray diffractometer (Panalytical Xpert Pro MPD) at room temperature with a monochromatic Cu kα radiation source (λ = 0.154 nm) with 2ϴ angle ranging from 10° to 50°, operated at a voltage of 45 kV and a current of 40 mA.

#### 2.5.6. Atomic Absorption Spectroscopy (AAS)

The initial amount of chromium and cadmium in the metal-ion solution was determined by acid digestion and subsequent AAS analysis. Firstly, the pharmaceutical effluent was subjected to AAS, which showed 4 heavy metals in it. Later, the amount of metal salt taken was 100 mg/kg, and after the remediation for 160 min the samples at specific intervals were sent for AAS analysis.

## 3. Results and Discussion

### 3.1. UV

**Figure 3:**
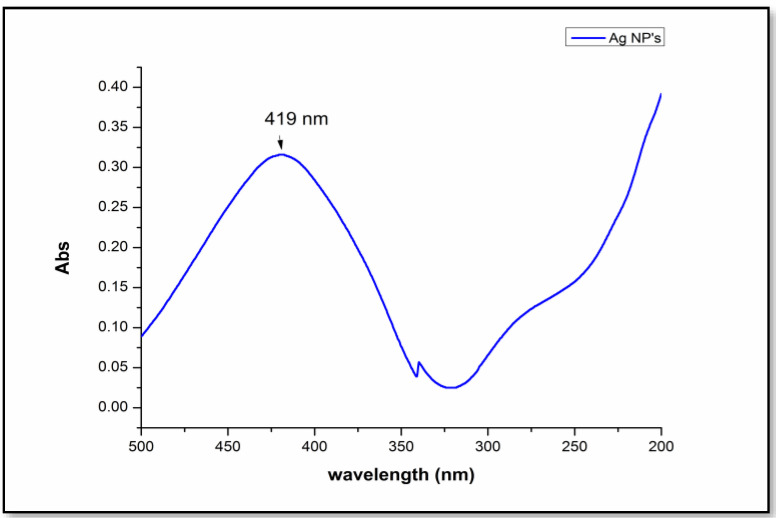
Absorption spectrum of silver nanoparticles synthesized from waste peel extract.

The reaction mixtures containing AgNO_3_ with orange peel extract turned yellow to brown after 12 h of incubation in the dark. Control silver nitrate solutions without the extract of orange peels did not develop a brown colour. This shows that the waste orange peels successfully acted as a reducing and capping agent. Plasmon resonance was displayed by silver nanoparticles. Figure 3 shows the absorption spectrum of silver nanoparticles at 419 nm, which confirmed its presence [30].

### 3.2. XRD

**Figure 4:**
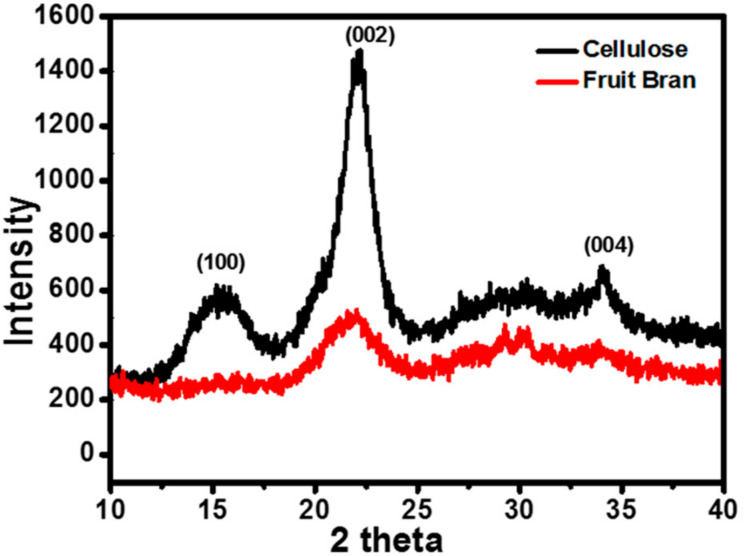
X-ray diffractogram of NFC and orange bran.

Orange bran and nano-fibrillated cellulose were analysed by XRD for their crystallinity index. Figure 4 shows X-ray diffractogram of isolated NFC and fruit bran. Nano-fibrillated cellulose showed a characteristic peak at 2ϴ = 29° and 2ϴ = 30°. Mostly, the diffraction angle lies between 25–35° for nanocellulose, which can be indexed as (100), (002), and (004) inferring to the monoclinic phase. This result is in accordance with the findings of Morais J and group [25]. Using X-ray diffraction data and Scherrers equation t = kλ/βCosϴ, the average crystallite size was estimated where *t* is crystallite size of the sample, β is full width half maxima (FWHM), and k is the wavelength of X-ray used, i.e., 1.548. The average grain size calculated was 6 nm. With the help of the intensity of amorphous peaks, the crystallinity index of orange peel bran and NFC was calculated. The crystallinity index is related to the strength and stiffness of fibres [31]. The crystallinity index of orange bran was 39% and that of NFC was 75%. Hence, the crystallinity index was increased by 36% after the chemical and mechanical treatments. It seems to be that the acid hydrolysis had much less effect on the morphology of these particles.

### 3.3. FTIR

**Figure 5:**
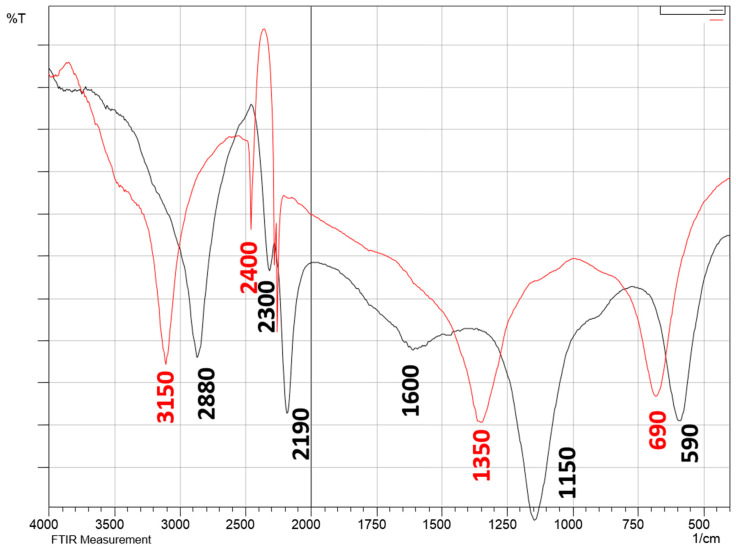
Comparative FTIR spectra of NFC and waste peel bran. —FTIR Spectra of waste peel Bran; —FTIR Spectra of lyophilised Cellulose.

The FTIR spectra of orange peel bran and nano-fibrillated cellulose are shown in Figure 5. Both spectra are dominated by peaks at 720 cm^−1^ and 3025 cm^−1^, which correspond to -C-H and = C-H stretching vibrations of hemicelluloses. The sharp band between 3000 and 3050 cm^−1^ shows -OH groups present and reflects the hydrophilic character of orange bran and nano-fibrillated cellulose. Aliphatic saturated C-H stretching vibration in NFC is seen in the peak at 2970 cm^−1^. The band at 1550 cm^−1^ demonstrates the bleaching step, which removed most of the lignin from nano-fibrillated cellulose. The peak at 760 cm^−1^ of C-H disappeared after NaClO_2_ treatment. The band near 1050 cm^−1^ is related to xylans, which was significantly less intense for the NFC sample. These features proved the β-glycosidic linkages between anhydro-glucose units in cellulose.

### 3.4. FESEM

**Figure 6:**
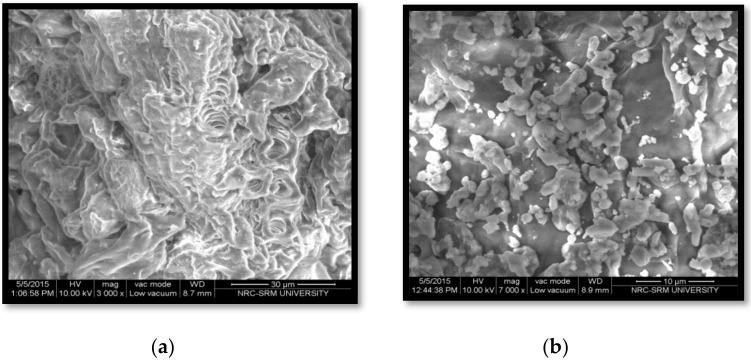
FE-SEM micrographs of waste peel bran (**a**) and NFC (**b**).

Figure 6a,b shows scanning electron microscopy micrographs of waste orange peel bran and NFC. The bran had an irregular surface and few grinding residues. Structural and chemical changes were depicted after the chemical treatment and morphological changes occurred in fibres with these steps. Amorphous components such as pectin and hemicelluloses were gradually removed.

The mineral acid employed in the hydrolysis step had a major influence on the surface properties of NFC. Additionally, the source of cellulose plays a wider role in the morphology and dimensions of NFC. Algal and tunicate cellulose produces nanocrystals of several microns, whereas wood fibres liberate shorter ones.

### 3.5. TEM

#### 3.5.1. Nano-Fibrillated Cellulose

**Figure 7:**
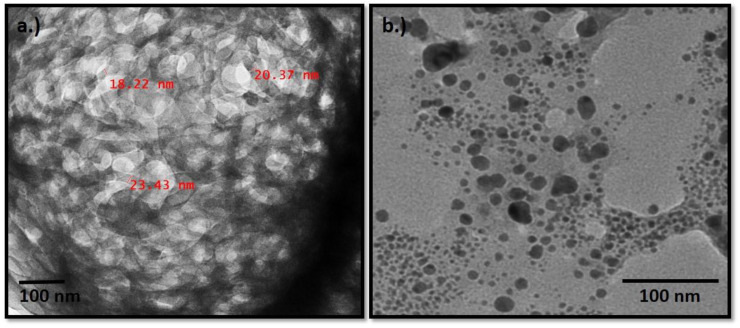
(**a**) TEM micrographs of nano-fibrillated cellulose (**b**) TEM micrograph of silver nanoparticles.

Figure 7a shows TEM micrographs of a diluted suspension of nano-fibrillated cellulose from waste orange peels. It can be seen that nano-fibrillated cellulose is mostly spherical and rarely oval. The majority of the overall size of particles lies in the nanometric range, i.e., 44–50 nm. Cellulose particles in amorphous regions are randomly overlapping each other and are spherical in shape. They have no irregular surface as they consist of amorphous and crystalline regions. Incomplete removal of hemicelluloses during chemical treatment and the formation of inter-fibrillar hydrogen bonds account for the presence of residual nanoparticle bundles [32].

#### 3.5.2. Ag Nanoparticles

Figure 7a of TEM confirmed the presence of silver nanoparticles. This study showed that the silver nanoparticles are spherical in shape and are polydispersed. The size of particles obtained was 30–34 at 25 °C. The synthesized silver nanoparticles from waste orange peels were stable in solution over a period of 3 months at room temperature [33].

### 3.6. AAS

**Figure 8:**
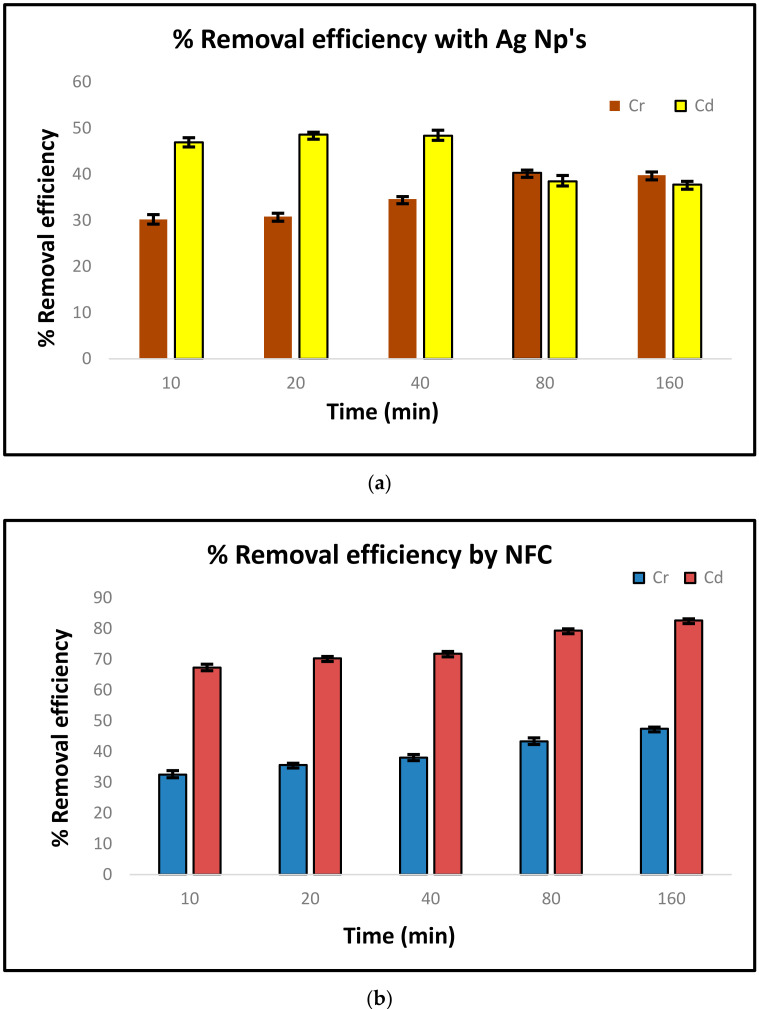

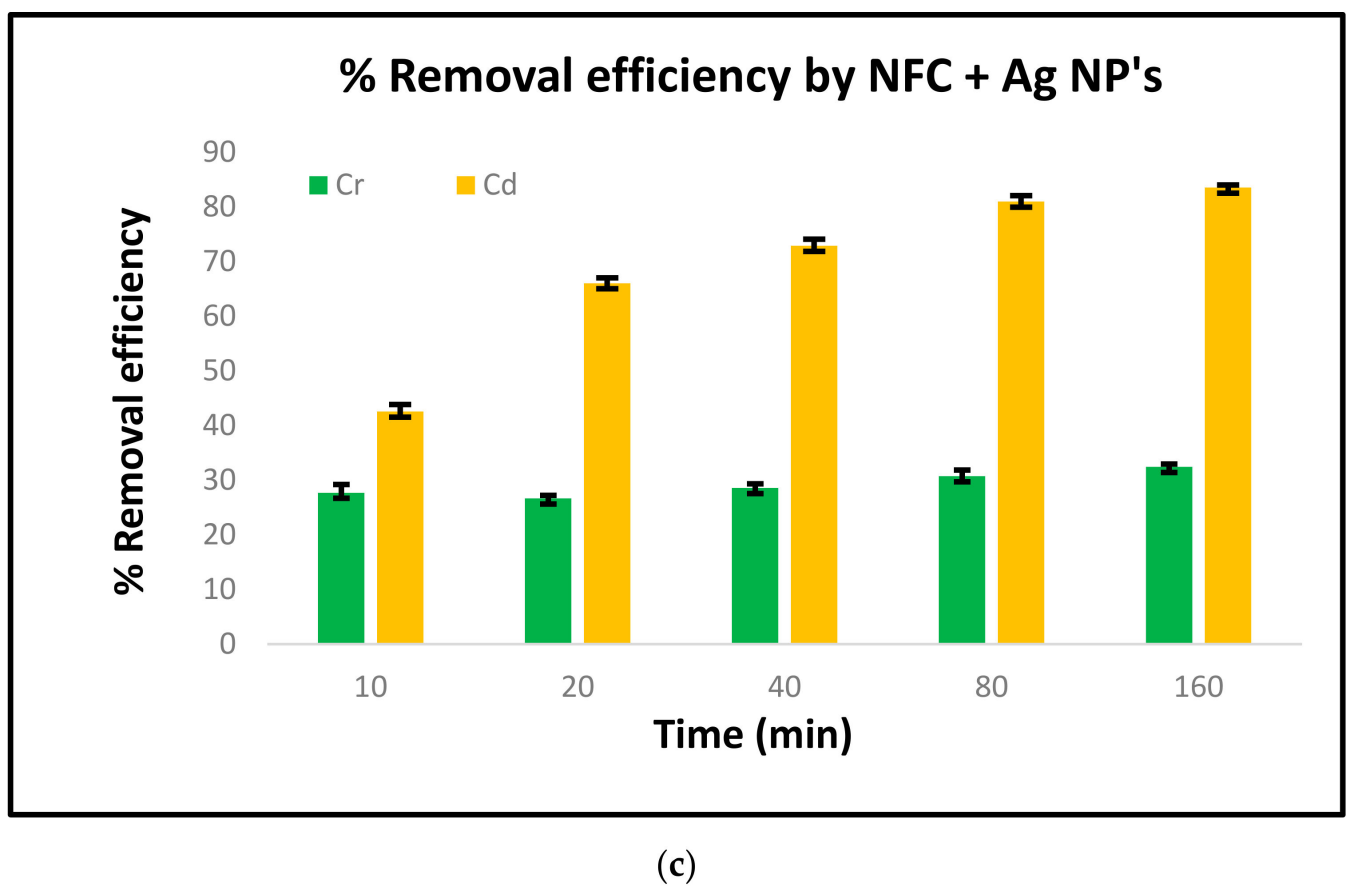
(**a**) Comparison chart of % removal efficiency in cadmium and chromium containing metal solution with silver nanoparticles at specific intervals. (**b**) Comparison chart of % removal efficiency in cadmium and chromium containing metal solution with NFC at specific intervals. (**c**) Comparison chart of % removal efficiency in cadmium and chromium containing metal solution with silver nanoparticles and NFC at specific intervals.

The removal efficiency at each interval was calculated by the formula (*C_i_* − *C_f_*)/*C_i_* × 100, where *C_i_* and *C_f_* are the initial and final metal concentrations, respectively. Figure 8a–c shows the comparative chart for the removal efficacy of Cr (II) and Cd (II). The highest removal efficiency was found for cadmium, i.e., 83.49%, by using silver and NFC together as a bio sorbent. The cellulose acted as an absorber, where it makes an interface with the silver nanoparticles and enhances the efficacy. The second highest was for cadmium, i.e., 47.21%, but by using only nano-fibrillated cellulose as a bio sorbent. The maximum sorption for both cadmium and chromium was observed with silver along with NFC, i.e., 83.49% and 32.20%, respectively. In one set with cadmium containing silver as a bio-sorbent, after 40 min the metal-ion estimation which reduced showed an increase of 10 mg/lit at 80 min. This was because the adsorbate and adsorbent attained equilibrium and were incapable of further sorption. Thus, at one point after equilibrium, concentration was lowered in the metal-ion concentration solution and, again, the substance undergoing the sorption changed to its bulk state [34,35].

### 3.7. Sorption Isotherms

**Figure 9:**
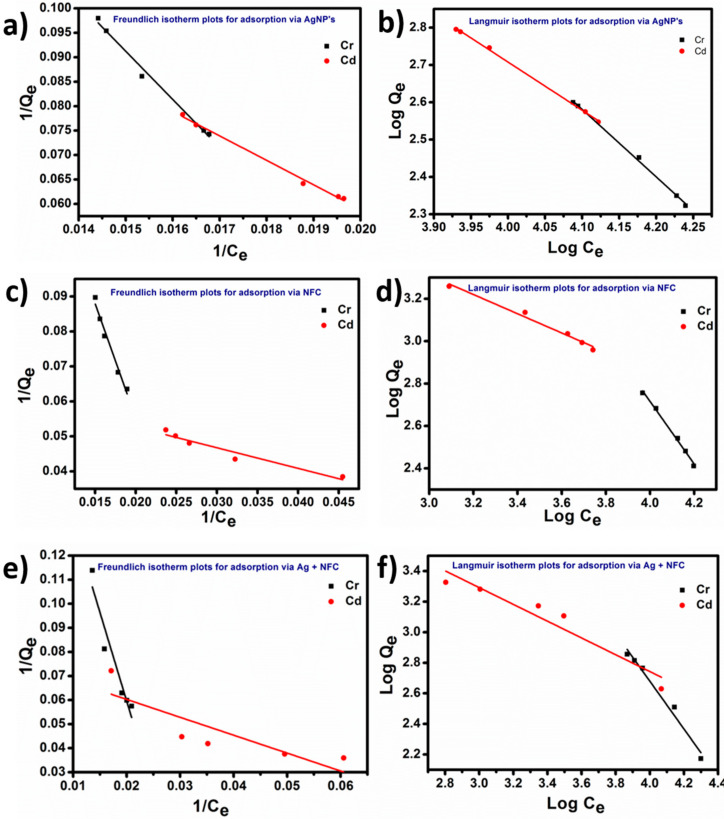
Freundlich and Langmuir isotherm kinetic simulation plots for Cd and Cr with three different adsorbents.

The maximum extent to which chromium and cadmium can perform sorption by NFC has been measured through the Langmuir and Freundlich isotherm. Figure 9a–f shows the kinetic simulation plots for Freundlich and Langmuir isotherms with three sets of adsorbent within 160 min. Due to wider gaps in the data obtained for Cd (II) and Cr (II), the linear plots have been separately visible on graph of each isotherm. The concentration was measured by taking sample for atomic absorption spectrometry at specific interval by doubling the time. So within 160 min, the concentration reduced up to ~17 ppm from 100 ppm in both the heavy metals. The kinetic plots for both isotherms with three adsorbents were fitted by using Origin 8.5. The linearity in the plots of Freundlich and Langmuir isotherms suggested the reaction mechanism followed first order rate kinetics.

As per the model designed by Freundlich, the overall optimum biosorption capacity (*K_f_*) was obtained for Cd (II): −0.74, while its intensity (1/*n*) was optimum for Cr (II): 0.21. *K_f_* and (1/*n*) were analyzed for the adsorbent mixture of AgNPs and NFC. This denotes higher efficiency for the sorption of divalent ions when combined together rather than utilizing bare AgNPs or bare NFC. Similarly, biosorption capacity (*Q*_0_) and energy (*b*) from Equation (5) of the Langmuir isotherm were found highest in energy for Cr (II): 8.92 while the biosorption capacity for Cd (II) was found to be −0.54. The appropriate high values of R^2^ inferred that both Freundlich and Langmuir isotherm models were fitted well to the ions of adsorbent Ag + NFC. The linear graphs pertained to the formation of a monolayer of Cd (II) and Cr (II) that may be homogenous [36].

## 4. Conclusions

The present study offers a simple and convenient method of synthesis of nano-fibrillated cellulose from agricultural residues of fruit. Experimental results showed that produced nanocellulose had a diameter within the range of 44–50 nm. FTIR showed the removal of lignin and hemicellulose due to the alkaline treatment. TEM confirmed the presence of nano-fibrillated cellulose, which showed a spherical shape of 44 nm. Silver nanoparticles were also synthesised and characterised using orange peel extract as a reducing agent. Their size was found to be 15–16 nm at 25 °C. The heavy metal ion removal efficiency was successfully analysed for cadmium and chromium by adding silver and nano-fibrillated cellulose at specific intervals. Thus, the present work showed the successful sorption activity of cadmium and chromium by nano-fibrillated cellulose with a removal efficiency of 83.49% and 32.20%, respectively. These can also be a possible commercial use for minimizing or removing toxic metal ions from water bodies.

## Data Availability

The data presented in this study are available on request from the corresponding author.
